# Industrial Fermentation of *Auxenochlorella protothecoides* for Production of Biodiesel and Its Application in Vehicle Diesel Engines

**DOI:** 10.3389/fbioe.2015.00164

**Published:** 2015-10-19

**Authors:** Yibo Xiao, Yue Lu, Junbiao Dai, Qingyu Wu

**Affiliations:** ^1^Ministry of Education Key Laboratory of Bioinformatics, Center for Synthetic and Systems Biology, School of Life Sciences, Tsinghua University, Beijing, China

**Keywords:** microalgae biodiesel, *Chlorella*, heterotrophic cultivation, cost assessment, diesel engine test

## Abstract

Microalgae-derived biodiesel has been regarded as a promising alternative for fossil diesel. However, the commercial production of microalgal biodiesel was halted due to its high cost. Here, we presented a pilot study on the industrial production of algal biodiesel. We began with the heterotrophic cultivation of *Auxenochlorella protothecoides* in a 60-m^3^ fermentor that produced biomass at 3.81 g L^−1^ day^−1^ with a neutral lipid content at 51%. Next, we developed plate-frame filter, natural drying, and ball milling methods to harvest, dry, and extract oil from the cells at low cost. Additionally, algal biodiesel was produced for a vehicle engine test, which indicated that the microalgal biodiesel was comparable to fossil diesel but resulted in fewer emissions of particulate matter, carbon monoxide, and hydrocarbon. Altogether, our data suggested that the heterotrophic fermentation of *A. protothecoides* could have the potential for the future industrial production of biodiesel.

## Introduction

We will eventually have to face the time when all fossil fuels, i.e., petroleum, natural gas, and coal, run out. Even before that time, climate change might destroy the entire planet if we continue to rely heavily on fossil fuels as we have, and we are now already experiencing warming of over 1°C (http://news.yahoo.com/not-run-fossil-fuels-op-ed-231243174.html). Recent advances in drilling and hydraulic fracturing technology to explore shale gas (Weber and Clavin, 2012) are exciting and may relieve concerns about fossil fuels for some time. However, even this resource may eventually be exhausted, as it is still not infinite. Meanwhile, concerns are rising due to its potential damage to the environment, especially land and habitat fragmentation, and its impact on air and water quality (Entrekin et al., 2011; Olmstead et al., 2013; Vidic et al., 2013). Therefore, there continues to be interest in developing new technologies to utilize other renewable and sustainable energy sources, such as solar, wind, geothermal, and biomass (Hidy, 2012).

Due to their faster growth rate, higher lipid content and less impact on food security than agriculture-based feedstocks, algae have attracted increasing amounts of attention for biofuel production in recent years (Brennan and Owende, 2010; Weyer et al., 2010). However, projections have largely depended upon small-scale experimental data. Most open pond systems and photobioreactors have been used to culture microalgae for high-value products but not for biodiesel due to the limitations of the lipid content under such cultivation conditions and the high cost for biomass (Rawat et al., 2013). To simultaneously achieve fast cell growth and high lipid content, the heterotrophic cultivation of *Auxenochlorella protothecoides* has been developed (Miao and Wu, 2006; Xiong et al., 2008; Ceron-Garcia et al., 2013). After optimization, the lipid productivity of *A. protothecoides* could reach 11.8 g L^−1^ day^−1^ (Xiong et al., 2010), much higher than that of photoautotrophic algae [0.2 g L^−1^ day^−1^ as reported by Rodolfi et al. (2009)]. It could, therefore, potentially be applicable for the commercial production of microalgal biodiesel. In addition, a previous study to evaluate microalgal biodiesel prepared from an 11-ton bioreactor suggested that it was comparable to conventional fossil fuels and complied with the US biodiesel standard (Li et al., 2007). Lately, many improvements at the lab scale for biodiesel preparation from algal lipids or directly from dry and wet cells using chemical or enzymatic approaches have been made to increase the efficiency of transesterification and to obtain high quality final products (Miao and Wu, 2004; Johnson and Wen, 2009; Levine et al., 2010). However, due to the difficulty in obtaining enough biomass for industrial production of microalgal biodiesel, only a few studies in recent years have been able to apply algal biodiesel to original or unmodified vehicle engines (Haik et al., 2011; Al-Lwayzy and Yusaf, 2013).

With the aim to produce microalgal biodiesel for commercial application, we report here the first large-scale trial of an algal biodiesel manufacturing and evaluation process, including the step-wise enlargement of biomass production, cell harvesting and drying, and biodiesel preparation by transesterification followed by quality assessment. After generating enough algal biodiesel for application tests, combustion experiments in a four-cylinder vehicle diesel engine were conducted, and several main features, including engine torque and emission, were monitored.

## Materials and Methods

### Microalga Strain and Basal Culture Medium

The microalga *A. protothecoides* strain was originally obtained from the Culture Collection of Algae at University of Texas (Austin, TX, USA) and screened in the Laboratory of Microalgae Fermentation and Bioenergy at Tsinghua University, Beijing, China. The basic culture media were the same as reported previously (Xiong et al., 2008).

### Production and Harvest of Microalga Biomass

**Figure 1:**
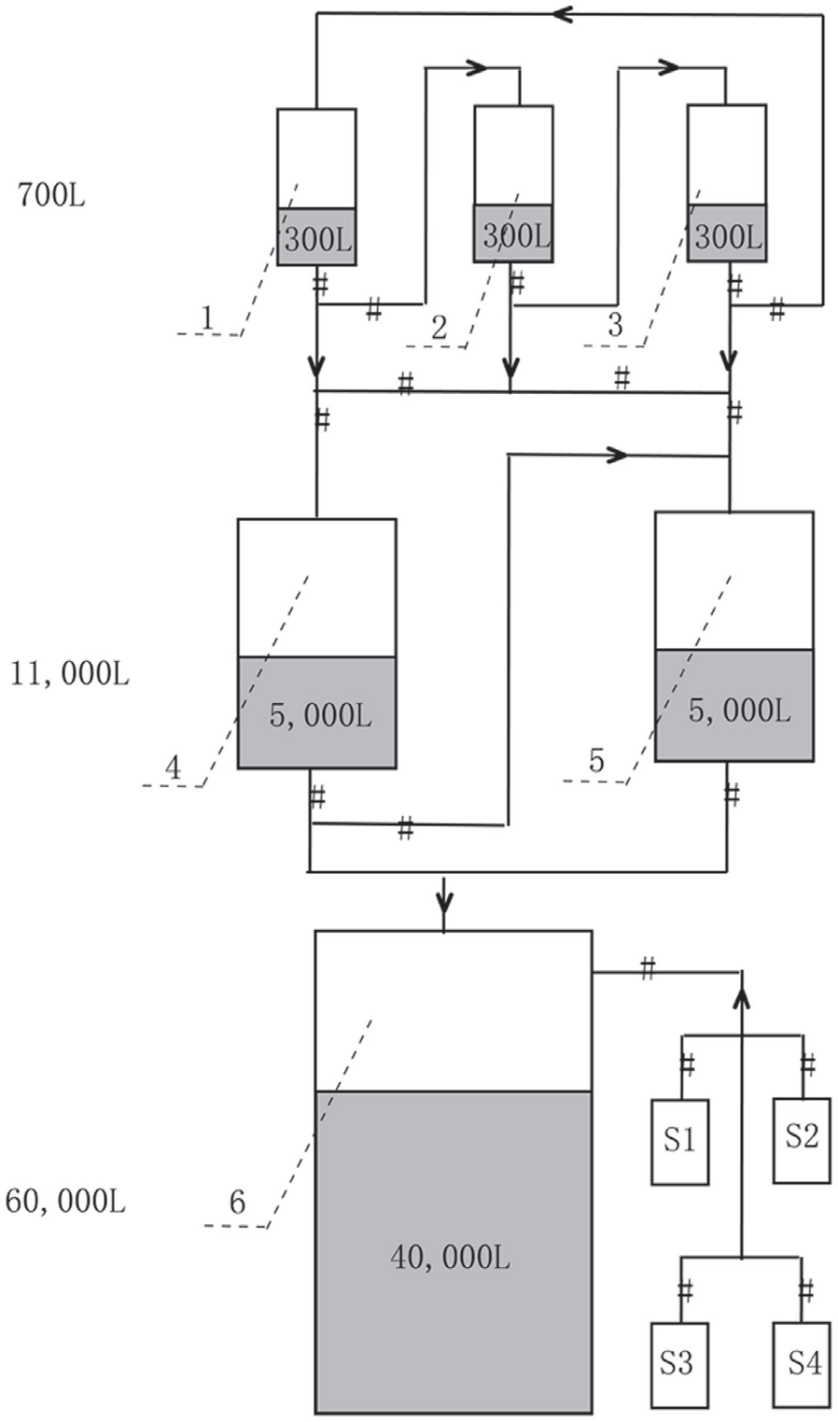
**Mass production strategy of heterotrophic *A. protothecoides* biomass in 700 L (Tanks 1–3, with 300 L medium each), 11 m^3^ (Tanks 4 and 5, with 5 m^3^ medium each), and 60 m^3^ (Tank 6, with 40 m^3^ medium) industrial fermentors**. S1–S4 are supplemental tanks for feeding glucose, nitrogen, NaOH, and antifoam, respectively.

The production of *A. protothecoides* biomass was carried out at North China Pharmaceutical Huasheng Co., Ltd., and the scaling-up fermentation was divided into three steps (Figure 1). First, the seed cultivation was performed in three 700 L stirred tank bioreactors containing 300 L basal medium with turbine impellers. Two liters of *A. protothecoides* cells at the exponential stage was inoculated into each fermentor, for which the temperature, pH, aeration rate, and agitation speed were initially set at 28 ± 0.5°C, 6.3 ± 0.1, 25 m^3^ h^−1^ (1:1 vvm), and 360 rpm, respectively. The pressure of the inner fermentor was kept at 0.04 Mpa except for the 60-m^3^ fermentor, which was kept at 0.2 Mpa. After 10 days of cultivation, the cells were partly inoculated into an 11-m^3^ fermentor containing 5 m^3^ of medium and kept at the same conditions as those of the 700 L fermentors. Finally, cells at the exponential stage from the 11-m^3^ fermentor were completely inoculated into a 60-m^3^ fermentor with 40 m^3^ of medium. We followed the optimized concentration of nitrogen and carbon source according to our previous published paper (Xiong et al., 2008). During fermentation, the cells were sampled every 6 h to determine their glucose concentration by the dinitrosalicylic acid (DNS) method (Miller, 1959) and the organic nitrogen concentration by the Nessler’s reagent method (Thompson and Morrison, 1951). Concentrated glucose solution (40%), corn steep liquor (0.3%), and antifoam solution (10%) were fed depending upon the substrate consumption. Five molar KOH was injected continuously to keep the pH at ~6.0.

### Cell Growth Estimation

To monitor the cell growth, 10 mL cell suspensions were collected. After centrifugation followed by twice washing with distilled water, the cells were oven dried until constant weight to determine the cell density (dry cell weight g L^−1^).

At the end of the fermentation, the cells were harvested by filtration at 0.05–0.12 MPa using plate-frame vertical pressure filters with 32 pieces of cloth for 12 h and were then spread on the open ground under sunlight for 2 days to dry. The water fraction of the algal biomass, represented by the moisture content (MC), was calculated as

(1) $$\text{MC}=\left(W_{\text{w}}-W_{\text{d}}\right)/W_{\text{w}}\times \text{1}00\%$$

where *W_d_* is the weight of the completely dried cells using a frozen vacuum dryer and *W_w_* is the weight of the biomass (*W_w_* − *W_d_* was the water left in the biomass).

### Oil Content Determination and Extraction

For the oil content (OC) determination, 10 g freeze-dried cells was hand-ground with silica in a mortar. The oil was extracted with *n*-hexane by the Soxhlet extraction method for 72 h. The *n*-hexane was subsequently evaporated, and the OC (weight of oil/weight of biomass × 100%) was calculated. For large-scale oil extraction, 30 kg dry cells, 30 kg steel grinding balls (3–5 mm in diameter) and 30 L isopropyl alcohol (IPA) were loaded into a 100-L steel ball mill machine (an industrial piece of equipment used in the food, oil and mining industries, XQM-100, Tianchuang Powder Equipment Co., Ltd., China) and ground at 200 rpm for 1 h. After recovering the milling balls using a stainless steel screen with a mesh size of 2 mm, the solution was filtered by a plate and frame filter. The oil-IPA miscella was vacuum distilled to recover the IPA. The crude oil was refined, and the oil extraction efficiency (OEE) was calculated as follows:

(2) $$\text{OEE}={\text{OW}}_{\text{x}}/\left({\text{BW}}_{\text{x}}\times \text{OC}\right)\times \text{1}00\%$$

where OW_x_ is the weight of the oil extracted with the ball milling process and BW_x_ is the weight of the dried biomass.

To investigate the effects of the ball mill machine on cell breakage and OEE, the microstructure of the broken cells (after oil extraction) was examined by scanning electron microscopy (Oberkochen, Germany). Both intact and broken cells were dehydrated sequentially with 50, 75, 90, and 100% ethanol (volume fraction) for 1 h each. After natural volatilization, the samples were spread on conductive tape, coated with gold powder and subjected to microscope examination.

### Preparation of Microalgal Biodiesel

The transesterification of the microalga oil was catalyzed by liquid lipase (12,000 U g^−1^, Sichuan Habio Bioengineering Co., Ltd., China) with the volume fraction of catalyzer:methanol (batch addition):oil at 1:10:100. The reaction was carried out in a 50-L bioreactor under constant stirring at 180 rpm and a temperature of 50°C for 8 h. The processes of methanol feeding and product separation were conducted according to a previous report (Li et al., 2007). After washing and distilling the remaining methanol, catalyst, and glycerol, the crude biodiesel at the top layer was rectified to obtain high quality biodiesel in a 100-L distillation tower with a maximum temperature at 220°C and vacuum degree at 0.1 Pa.

### Components and Quality Analysis of Algal Biodiesel

The biodiesel components were analyzed by gas chromatography–mass spectroscopy (GC–MS) (Thermo, USA). A dual-stage quadrupoles GC apparatus was equipped with a Varian VF-5 ms column (30 m × 0.25 mm ID DF = 0.25 μm), and the GC was manipulated with a flow rate at 10 mL min^−1^. The physical and chemical properties of the biodiesel were determined according to ASTM D6751 standards at the Institute Analysis Center, SINOPEC CORP. The petroleum diesel (PD) on the market was used for comparison.

### Vehicle Diesel Engine Test

Three types of fuel samples (100 L each) including 100% algal biodiesel (MBD100), 20% algal biodiesel blended with 80% PD (MBD20), and 100% PD were fueled and tested separately in a 3.0 L vehicle diesel engine (ZD-30, Nissan Motor Company, Japan) for their dynamic properties and emissions. The stationary bench tests were conducted in the National Key Laboratory of Automotive Engineering in Tsinghua University, Beijing, China. To apply load on the vehicle diesel engine, a dynamometer (GW300, Hunan Xiang Yi Dynamic Test Instrument Co., Ltd.) was used. The dynamometer was modified by adding a load cell and digital monitor to measure the engine speed (r min^−1^) and torque (Nm) to calculate the power (kW). The engine thermal efficiency was calculated as follows:

(3) $$\eta =\text{3}.\text{6}\times \text{1}0^{\text{6}}/\left(b\times H\right)$$

where η, *b*, and *H* are the engine efficiency, brake-specific fuel consumption (BSFC), and heat value of the fuels, respectively.

A gas analyzer (BEA 460 Bosch, Combustion Products Group, Ltd., UK) was used to monitor the engine exhaust gases for carbon monoxide (CO), hydrocarbon (HC), and nitrogen oxides (NOx). Particulate matter (PM) was measured by a 5–1000 nm particulate analyzer (DMS500, Combustion Products Group, Ltd., UK). Prior to the test, the device was subjected to maintenance and calibration by the manufacturer followed by daily standard calibration.

## Results

### Production of Heterotrophic *A. protothecoides* Biomass

**Figure 2:**
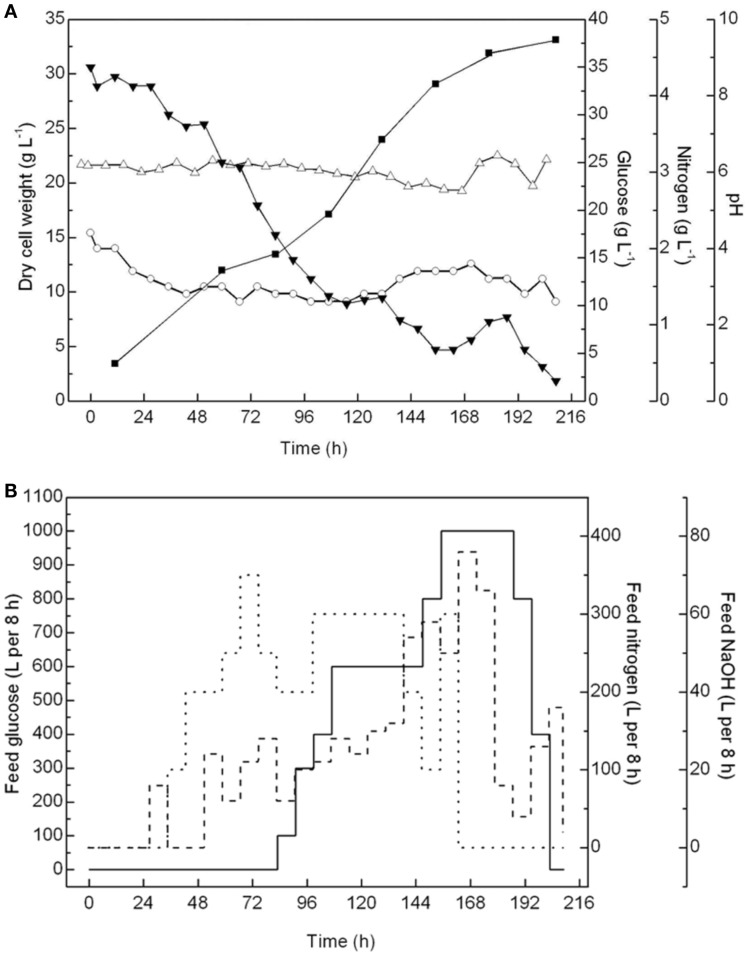
**Monitor and control of fed-batch cultivations of *A. protothecoides* in a 60-m^3^ fermentor**. **(A)** Time courses monitor records of cell growth (■), pH variation (Δ), residential concentration of glucose (▾), and nitrogen (○). **(B)** Time course profiles of feeding glucose (—), nitrogen (…), and NaOH (- - -).

The cell growth, pH, and glucose and nitrogen content in the 60-m^3^ fermentor were monitored throughout the fermentation process, and concentrated glucose solutions (40%), corn steep liquors (0.3%), and antifoam solutions (10%) were batch-fed accordingly (Figure 2). The pH was controlled at ~6.0 ± 0.2 to ensure optimal cell growth conditions. The amount of glucose and residual nitrogen in the container, which are critical to maintain the heterotrophic growth of *A. protothecoides*, were carefully controlled. According to our previous trials (data not shown), when cells escaped from the lag phase, a low residual concentration of glucose below 10 g L^−1^ in the culture medium could avoid the inhibition of cell growth, especially when both the glucose consumption rate and glucose feeding rate were high. Therefore, after 84 h, when the glucose content dropped to near 10 g L^−1^, the glucose feeding was initiated but was carefully regulated to ensure a residual concentration between 5 and 10 g L^−1^. Meanwhile, the amount of residual nitrogen was maintained at ~1.5 ± 0.2 g L^−1^, enough for the cells to proliferate.

After 216 h of fermentation in the 60-m^3^ fermentor, the final cell density reached 33.14 g L^−1^, with an average biomass productivity at 3.81 g L^−1^ day^−1^. Notably, before the cell density reached 30 g L^−1^ in the initial 144 h, the biomass productivity was above 6.87 g L^−1^ day^−1^. However, the cell growth was greatly inhibited after that, resulting in the biomass productivity in the last 48 h being <2.5 g L^−1^ day^−1^. This result suggested that under this fermentation condition, the cells may enter a stationary phase once the density is over 30 g L^−1^. Therefore, it becomes uneconomical to continue the fermentation process. In the future, we can either stop this fermentation or initiate the semi-continuous fermentation process at this time point. The biomass productivity in this 60-m^3^ fermentation was more than twice that (1.71 g L^−1^ day^−1^) in an 11-m^3^ fermentor in a previous study and 10 times more than that in outdoor photobioreactors (on average 0.30 g L^−1^ day^−1^) (Rodolfi et al., 2009).

### Sugar-Lipid Conversion Ratio in a 60-m^3^ Fermentor

The total glucose consumed in the 60-m^3^ fermentation was 5120 kg, and the yield of dry biomass was 1540 kg, indicating that the conversion ratio of glucose to biomass was ~0.301 kg/kg. Within these cells, the OC was determined to be 51%. Therefore, the total oil yield per fermentation was 739.5 kg, the oil productivity was 82.16 kg day^−1^, and the conversion ratio of glucose to oil was 0.154 kg/kg. However, during the oil production through carbon metabolism, glucose is first converted to pyruvate via glycolysis and is then transformed to acetyl-CoA. The theoretical maximum conversion ratio of glucose to oil is 51.1% (mass fraction, i.e., 66.7% mole fraction) (Xiong et al., 2010). However, a previous study indicated that the conversion ratios in 5 L and 11 m^3^ fermentors were 31.2 and 27%, respectively (Li et al., 2007), suggesting that the increased volume may reduce the conversion rate. Additional studies should be performed to increase the conversion rate, especially in larger fermentors.

### Algal Harvest, Drying, and Oil Extraction

The processes to harvest and dry the microalgae biomass and to extract oil from the cells are the other important energy- and labor-consuming steps in microalgal biofuel production and could account for a significant fraction of the total cost (Islam et al., 2014). In the laboratory, centrifugation is commonly used to collect the algal biomass followed by freeze or spray drying (Xu et al., 2011; Wang et al., 2012), and the method of Soxhlet was generally used to extract oil (Yan et al., 2011; Mohan and Devi, 2014). However, these methods require either high-cost equipment or high energy consumption, and their processing capacity is only limited to a small amount of biomass. Therefore, it is not feasible to apply these methods in commercial production.

**Figure 3:**
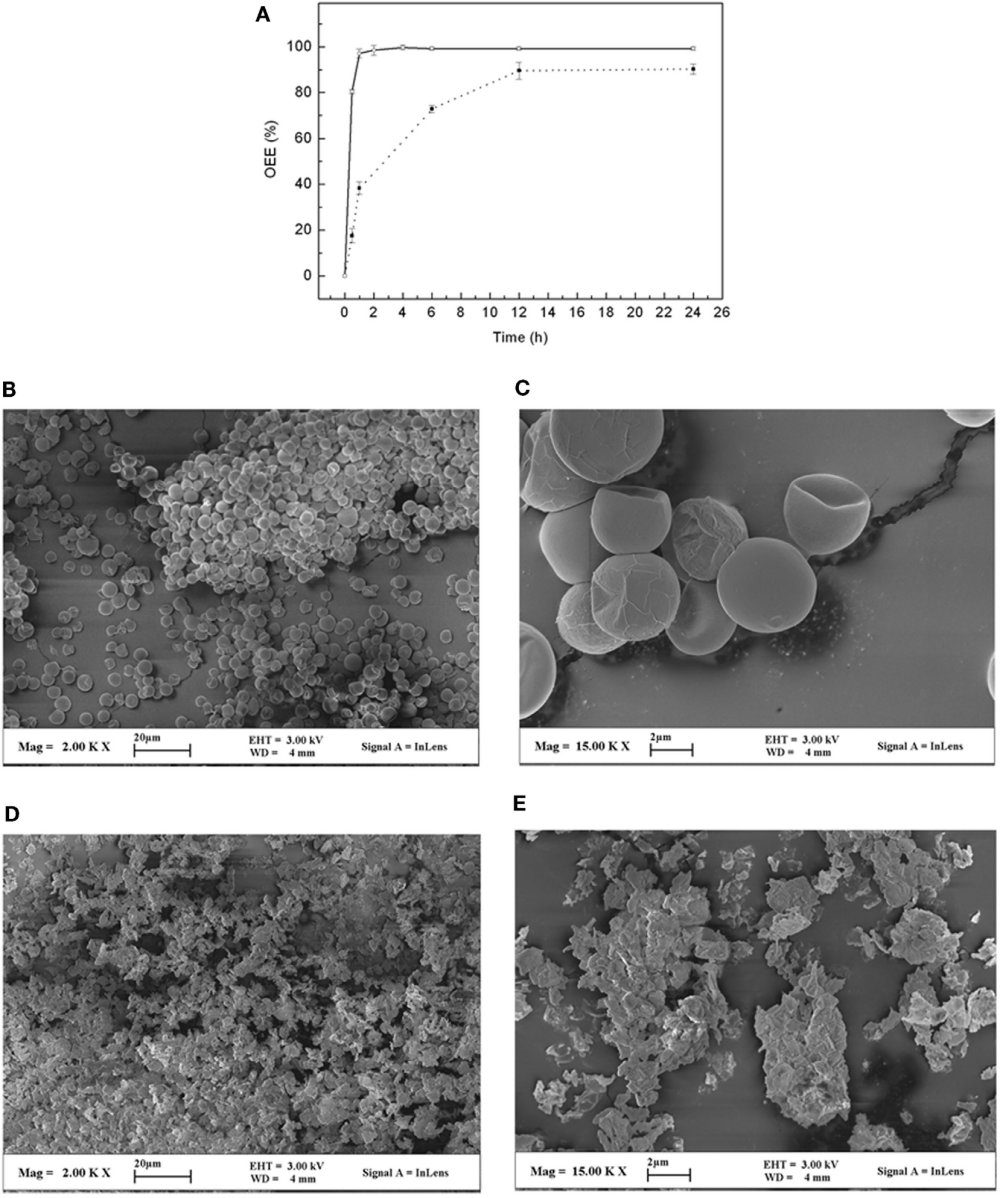
**Oil extraction from *A. protothecoides* cells by ball milling with IPA as solvent**. **(A)** Effects of ball mill time on oil extraction efficiency (OEE) of algal biomass with MC at 5% (○) and 66% (■). **(B,C)** Photos of scanning electron microscopy of *A. protothecoides* cells before ball milling at small **(B)** and large **(C)** magnifications, see scale bar. **(D,E)** Photos of scanning electron microscopy of cell residues after ball milling with IPA at small **(D)** and large **(E)** magnifications, see scale bar.

To handle the large volume of final fermented suspension from a 60-m^3^ fermentor and simultaneously avoid the high energy and high-cost centrifugation process, we developed a filtering method that enables us to obtain wet biomass by gravity supplemented with vertical pressure (0.05–0.12 MPa) through the plate-frame filter with 32 pieces cloth (a patent filter device, Chinese patent application No. 201310285550.8). This method could recover ~98% of algae, and the medium after filtering could theoretically be recycled and used for another round of fermentation. The MC showed that the wet biomass from the plate-frame filter contained 66% water, which could be further dried. In addition, there are several recent reports on direct oil extraction using wet biomass (Islam et al., 2014), and similar processes could potentially be applied here. Meanwhile, we tested two different methods to dry the cells. One method is using a commercial oven to bake the wet algae, and the other is to simply expose the wet biomass on the open ground under natural sunlight. We found that both methods worked well by reducing the MC to 0 or 5% after 48 h, respectively. However, when the cells were taken out of the oven, they formed a solid plaque that was very hard to break and prevented the subsequent oil extraction process. However, the cells dried under natural sunlight remained as a soft powder and could be easily processed. In addition, natural drying also avoided an extra electricity cost, and we, therefore, think it is a better method for cell drying. Furthermore, we also tested if the naturally dried cells could potentially affect the oil extraction process and found no difference between normal dried cell samples (Figure 3A). Overall, the energy consumption for the plate-frame filter was calculated as 0.014 MJ kg^−1^. Compared with that using centrifuge and freeze or spray drying, which required at least 80.9 MJ kg^−1^ (Xu et al., 2011), the method presented in this study was economical and feasible for industrial application.

Oil extraction from algal cells is another challenge for the commercial production of biofuel. The laboratory-scale Soxhlet method is not applicable, as it could only deal with a very small amount of dried cells (generally <100 g) and took a long time (generally 3 days). To overcome this problem, we tested whether oil could be directly extracted by organic solvent when cell disruption was performed simultaneously in a 100-L steel ball mill machine, which allowed us to process a biomass of ~360 kg/day. Considering the lipid composition of heterotrophic *A. protothecoides* and also safety issues, IPA was used due to its high boiling point and low toxicity. As shown in Figure 3A, the OEE was 65.89 ± 1.31% after immersing the dry algal cell (MC at 2%) in IPA for 1 h without physical disruption. However, when the cells were subjected to ball milling in IPA, the OEE increased up to 97.12 ± 1.84%. In addition, we also tested whether we can directly extract oil from the aforementioned wet algal cells (MC at 66%). We found that it required a longer processing time and had a lower OEE than when using the dried cells (Figure 3A). To further confirm the effect of the ball milling method, a scanning electron microscope (SEM) was used to evaluate the cell integrity. We found that before ball milling, all algal cells were intact but shriveled due to dehydration (Figures 3B,C). After ball milling, the cells were almost completely crushed into tiny fragments (<0.2 μm) (Figures 3D,E). Most of the oil and intracellular constituents were taken into the IPA.

### Properties of the Algal Biodiesel

Given that enough crude oil could be obtained through above processes, we next carried out enzymatic transesterification to produce algal biodiesel and analyzed its composition. We found that the overall quality of the biodiesel was similar to what has been previously reported (Miao and Wu, 2006). A comprehensive evaluation of the derived algal biodiesel was conducted by comparing its chemical and physical properties with PD and the ASTM D6751 Standard. As shown in Table 1, the algal biodiesel was characterized by a flash point of 160°C, viscosity of 4.354 mm^2^ s^−1^, density of 876.9 kg m^−3^, acid number of 0.2, and heat value of 38.25 MJ kg^−1^. These properties satisfied most ASTM D6751 standards, except that the oxidation stability exceeded the designated range and could be improved (Schober and Mittellbach, 2004). The cetane number at 52.6 indicated that the microalgal biodiesel would combust better than number 0 fossil diesel. Overall, our study suggested that the microalgal oil derived from cells in a 60-m^3^ industrial fermentor could be used as feedstock for the production of high quality biodiesel.

**Table 1:** **Comparison of the properties of microalgae biodiesel, 0# petroleum diesel, and ASTM biodiesel’s standard**.

Properties	Unit	Microalgae biodiesel	0# petroleum diesel^a^	ASTM D6751 Standard
Flash point	°C	160	75	≥130.0
Viscosity	mm^2^ s^−1^	4.354	1.9–4.1	1.9–6.0
Density	kg m^−3^	876.9	838	820–900
Acid number	mg(KOH) g^−1^	0.2	≤0.5	≤0.50
Cetane number	–	52.6	40–51	≥47
Copper strip corrosion	Class	1	1	≤1
Cold filter plugging point	°C	1	≤4	Report
Heat value	MJ kg^−1^	38.25	40–45	Report
90% recovery temperature	°C	341.0	NA	≤360
Oxidation stability	h	1.2	NA	≥3
Water content	mg kg^−1^	26	NA	≤50
Sediments	–	None	None	None
Sulfated ash	% Mass	0.005	NA	≤0.02
10% Carbon residue	% Mass	0.09	NA	≤0.3
H/C ratio	% Mass	1.81	1.81	Report
Total glycerol content	% Mass	0.02	NA	≤0.24
Oxygen content	% Mass	10.66	NA	Report
Sulfur content	% Mass	8.3 × 10^−6^	0.95	≤0.02

*The data for diesel fuel are from published studies; NA indicates not available*.

### Application of Microalgal Biodiesel for Vehicle Engine Operation

Biodiesel has been tested in direct-injection engines or diesel tractors. For example, Dorado et al. (2003) tested biodiesel from olive waste cooking oil in a direct-injection engine. For microalgae-derived biodiesel, a recent report (Al-Lwayzy and Yusaf, 2013) suggested that it could satisfy the requirement in a diesel agriculture tractor engine, in which 20% microalgal biodiesel was used. However, there are still no reports if pure microalgal biodiesel (100%) could be used and how it will perform in a direct-injection engine.

**Figure 4:**
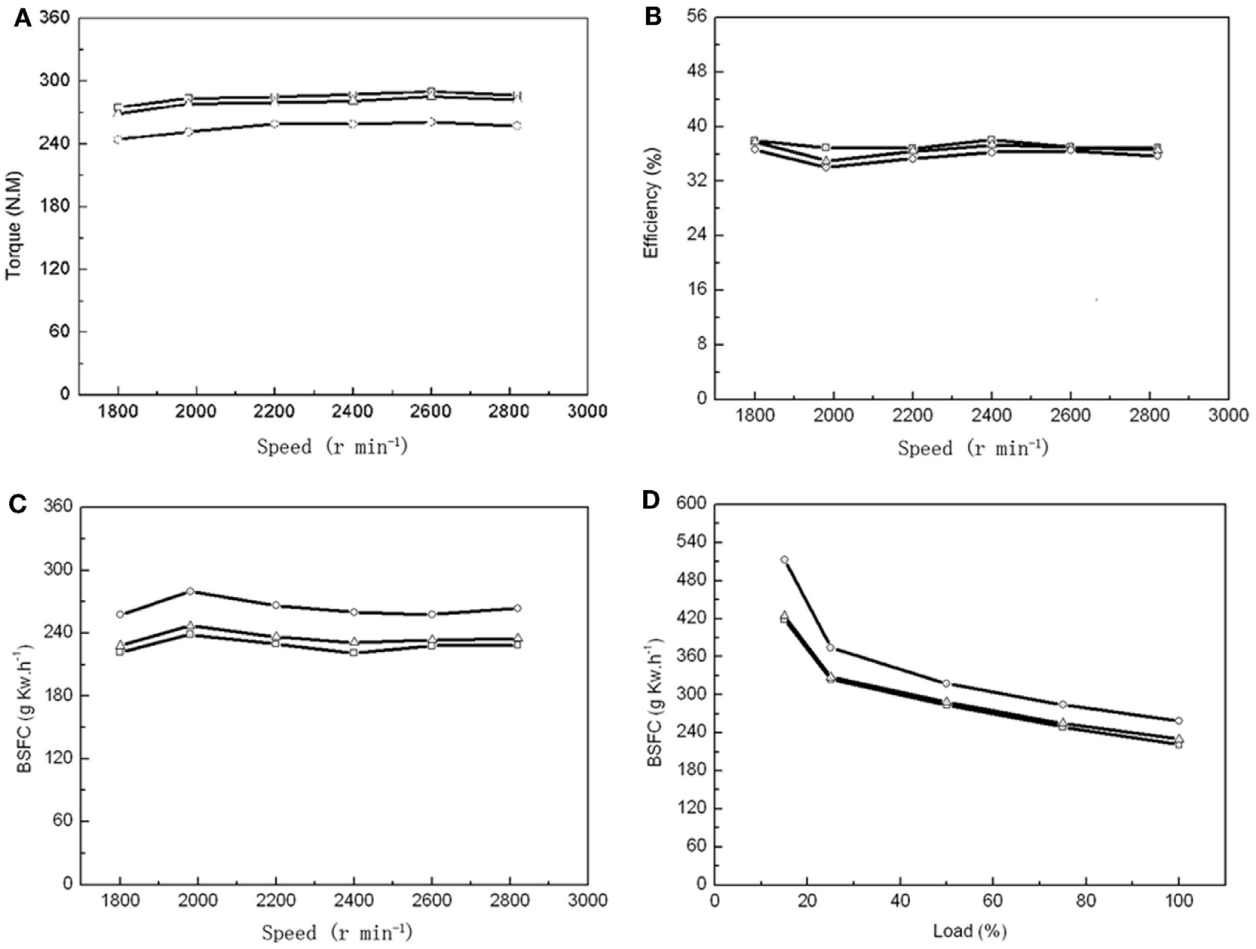
**Performance of vehicle diesel engine using petroleum diesel (PD, 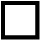), 20% microalgal biodiesel blend with 80% PD (MBD20, **Δ**), and pure microalga biodiesel (MBD100, ○), respectively**. **(A)** The relationship between engine torque and engine speed. **(B)** The relationship between engine efficiency and engine speed. **(C)** The relationship between engine brake-specific fuel consumption (BSFC) and engine speed. **(D)** The relationship between engine BSFC and engine load.

As a single 60-m^3^ fermentor could produce 1540 kg dry biomass with an OC over 50%, it enabled us to prepare enough algal biodiesel for 24 h combustion to test multiple parameters. Number 0 diesel, 20% microalgal biodiesel (B20), and 100% microalgal biodiesel (B100) were tested in a vehicle diesel engine to compare the BSFC, engine torque, thermal efficiency, NOx and PM emissions between these different fuels. The maximum torques was found at an engine speed of 2600 r min^−1^ for PD, MBD20, and MBD100 (Figure 4A). The engineering efficiency did not change significantly among PD, MBD20, and MBD100 at different speed (Figure 4B). The average dynamics reached ~98 and 90% of the peak values with MBD20 and MBD100, respectively, indicating no significant differences between PD and MBD20 at a speed of 2600 r min^−1^ at the peak torque. The BSFC (Figures 4C,D) was observed to increase with a higher proportion of algal biodiesel in the blend compared to PD at all engine speeds. No significant difference was found between PD and MBD20 in the entire load range, suggesting that MBD20 could be an optimal biodiesel blend.

**Figure 5:**
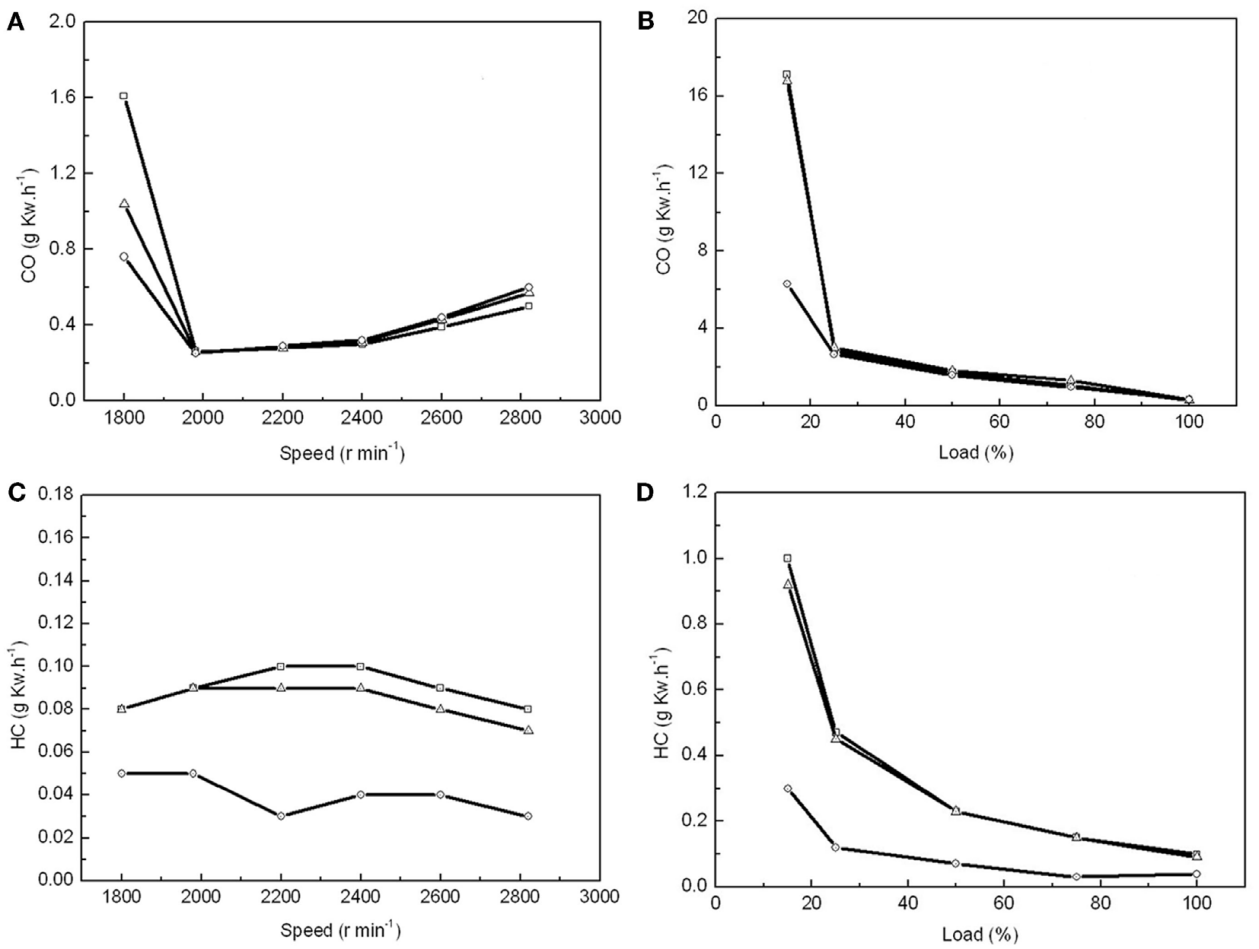
**The effects of combustion with PD, MBD20, and MBD100 on emissions of CO and HC**. **(A)** CO emission from different engine speeds. **(B)** CO emission at different engine loads. **(C)** HC emission at different engine speeds. **(D)** HC emission at different engine loads. PD (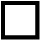), MBD20 (Δ) and MBD100 (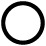).

Emissions of gas and PM are the other important parameters to evaluate in biodiesel. When the air supply was insufficient at a low engine speed and load, carbon monoxide (CO) was the main pollutant in the exhaust gas. We found that the CO emissions were 35 and 53% lower from the combustion of MBD20 and MBD100, respectively, than that from PD when the engine speed was below 1900 r min^−1^ and the engine load was below 20% (Figures 5A,B). This indicates better combustion for MBD20 and MBD100, which potentially resulted from the extra oxygen in the biodiesel. With increasing engine speed up to 2000 r min^−1^ or engine load up to 25%, the CO emission decreased greatly and did not subsequently show differences among PD, MBD20, and MBD100 (Figures 5A,B).

Hydrocarbon, as one of the main greenhouse gases that negatively impacts the formation of ozone, is another pollution gas from fuel combustion. The lowest HC emission at 0.03 g k Wh^−1^ was recorded at 2200 r min^−1^ for MBD100, which was only one-third of that for PD (Figure 5C). HC emissions for MBD20 were close to PD, which decreased significantly with increasing engine load (Figure 5D). In general, MBD100 produced less HC did PD and MBD20 under most conditions.

**Figure 6:**
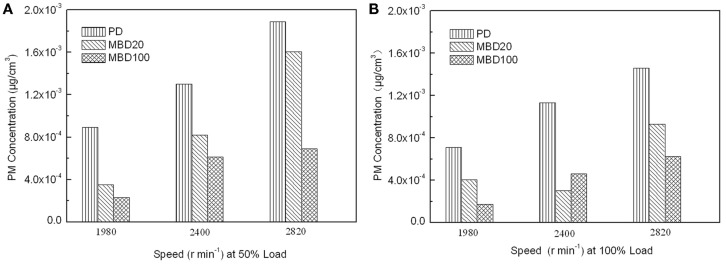
**The effects of combustion with PD, MBD20, and MBD100 on emissions of particulate matter**. **(A)** PM emission at different engine speeds with 50% engine load. **(B)** PM emission at different engine speeds with 100% engine load.

In addition, NOx, including NO and NO_2_ as pollution gases from fuel combustion, were detected in the engine test. No significant difference was observed among BMD 100, BMD 20 and PD for the test fuels (data not shown). In contrast, the PM emissions detected showed that the PM emissions from both MBD20 and MBD100 were obviously lower than those of PD under different engine speeds and loads (Figure 6). Compared with PD, the PM emission reduced from 37 to 42% in the MBD20 test and 57 to 75% in the MBD100 test, respectively. As a bio-oil derived fuel, the 10.66% oxygen content in the biodiesel molecules and the low sulfur content (mass fraction of 8.3 × 10^−6^) may contribute to a complete fuel oxidation, causing a significant decrease in the PM concentration. When the engine load doubled or the speed increased, the PM emissions were clearly reduced (Figure 6) as the formation of soot from the exhaust emission was proportional with the change of combustion temperature.

## Discussion

At the moment, it is still not economically viable to industrially produce microalgal biofuel, despite the potential for microalgae to be a sustainable and green bioenergy resource, largely because of limitations on microalgal productivity (Pienkos and Darzins, 2009; Davis et al., 2011). Several technologies have been developed to reduce the cost, including strain improvement, cultivation optimization, and chemical treatment (Specht et al., 2010; Scranton et al., 2015). Here, we extended the heterotrophic cultivation of *A. protothecoides* for biomass production from a laboratory to an industrial scale (Miao and Wu, 2006; Xu et al., 2006; Li et al., 2007). A 60-m^3^ fermentor of *A. protothecoides* was established, which produced microalgal biomass at 3.81 g L^−1^ day^−1^ on average and an OC of ~51%, comparable to what we previously obtained on a smaller scale (Miao and Wu, 2006; Xu et al., 2006; Li et al., 2007).

Plate-frame filter, natural drying, and ball milling methods were developed as subsequent energy-saving processing technologies. The plate-frame filter and natural drying procedure could replace the commonly used, high-cost centrifugation, which requires not only a large investment in equipment but also significant energy consumption. In addition, our study suggested that the ball mill disruption method could also be a feasible and scalable process to meet the industrial requirement for oil extraction besides its application in the recovery of bioproducts from cyanobacteria (Balasundaram et al., 2012). Compared with high-pressure single solvent (hexane) extraction (Islam et al., 2014), ball mill disruption consumes less energy and has low requirements for equipment.

Subsequently, the vehicle diesel engine test indicated that the microalgal biodiesel is comparable to fossil diesel but produces fewer emissions of PM, carbon monoxide, and HC emissions. Two types of biodiesel (MBD 20 and MBD 100) performed well and resulted in an acceptable increment of the volumetric fuel consumption in an unmodified vehicle diesel engine with fewer emissions of carbon monoxide, HC, and PM, which could potentially reduce damage to the environment.

Finally, based on a previous study on the commercial production process of biodiesel, the cost of crude oil feedstock contributes to 88% of the overall production cost (Haas et al., 2006). Accordingly, the cost of our microalgal biodiesel could be estimated at 18.73 US$/kg, which is equivalent to 15.70 US$/L (density of algal biodiesel measured at 0.838 kg/L). Although the minimum production cost of algal biodiesel in this study was higher than the current petrol diesel price in China (1.2 US$/L), it was much lower than the reported microalgae biodiesel produced from an outdoor photobioreactor (73.5 US$/L) (Lam and Lee, 2014), suggesting that the fermentation process could become competitive in the future once the production costs are further reduced. Several approaches could be adapted to cut the costs in the future, including (1) the substrate (glucose) feeding and process control in the fermentor can be further optimized to obtain a higher cell density and OC, reduce the fermentation period or improve the actual conversion ratio from sugar to oil; (2) the cost for the glucose substrate might be reduced by using other feedstocks, such as cassava (Lu et al., 2010), Jerusalem artichoke (Cheng et al., 2009), or waste molasses (Yan et al., 2011); (3) the off-gas from the fermentor could be recycled to agitate the medium in the photobioreactor and provide CO_2_ for photoautotrophic cells, which can be coupled with heterotrophic fermentation to address carbon reuse and cost reduction (Lu et al., 2013); (4) replacing the current 60-m^3^ stirring fermentor with an airlift bioreactor could save on electricity consumption for driving the big agitator blade; (5) a continuous fermentation process could be used to shorten the fermentation period and cut down on the corresponding consumption of electricity and aseptic air; and (6) high-value byproducts or co-products, such as carotene and lutein, might be explored to supplement algal biodiesel production.

## Conflict of Interest Statement

The authors declare that the research was conducted in the absence of any commercial or financial relationships that could be construed as a potential conflict of interest.
